# Overall and Relative Survival After a Second Contralateral Hip Fracture in Adults over 65 Years: A Retrospective Cohort Study

**DOI:** 10.3390/geriatrics11040083

**Published:** 2026-07-10

**Authors:** Maria-Dolors Rosinés-Cubells, Mercè Castejón, Joan Espaulella-Panicot, Andrew Ore-Zuñiga, Anna Arnau

**Affiliations:** 1 Department of Orthopaedic Surgery and Traumatology, Althaia Xarxa Assistencial Universitària de Manresa, 08243 Manresa, Spain; 2 Doctoral Program in Medicine and Biomedical Sciences, Universitat de Vic-Universitat Central de Catalunya (UVIC-UCC), 08500 Vic, Spain; 3 Institut de Recerca i Innovació en Ciències de la Vida i de la Salut a la Catalunya Central (IRIS-CC), 08500 Vic, Spain; 4 Faculty of Medicine, Universitat Internacional de Catalunya (UIC), 08195 Sant Cugat del Vallès, Spain; 5 Unitat Funcional de Fractures de Fèmur (UF3), Althaia Xarxa Assistencial Universitària de Manresa, 08243 Manresa, Spain; 6 Geriatric and Palliative Care Service, Fundació Hospital de la Santa Creu de Vic, Consorci Hospitalari de Vic, 08500 Vic, Spain; 7 Central Catalonia Chronicity Research Group (C3RG), Institut de Recerca i Innovació en Ciències de la Vida i de la Salut a la Catalunya Central (IRIS-CC), 08500 Vic, Spain; 8 Faculty of Medicine, Universitat de Vic-Central de Catalunya (UVIC-UCC), 08500 Vic, Spain; 9 Research, Epidemiology and Biostatistics Unit, Althaia Xarxa Assistencial Universitària de Manresa, 08500 Manresa, Spain

**Keywords:** hip fractures, survival, frailty, aged, functional status, mental status

## Abstract

**Background/Objectives:** The main objective of this study was to estimate 1- and 3-year overall survival and relative survival in patients aged >65 years who underwent surgery for a second contralateral hip fracture. **Methods:** Retrospective cohort study including patients aged ≥65 years who underwent surgery for a first or second contralateral hip fracture between June 2010 and December 2021. Overall survival (OS) was estimated from the date of surgery to death from any cause or end of follow-up. Relative survival (RS) was calculated as the ratio between observed survival and expected survival derived from general population mortality tables for Catalonia implemented in the WebSurvCa application. **Results:** A total of 2642 patients were included (2467 primary fractures; 175 s contralateral fractures). Patients with a second fracture were older and had worse pre-fracture functional and cognitive status. At 3 years, overall survival was 53.8% (95% CI: 51.7–55.9) after a first fracture and 43.7% (95% CI: 36.3–52.4) after a second contralateral fracture. Relative survival estimates were 70.5% (95% CI: 67.7–73.3) and 59.0% (95% CI: 49.1–70.8), respectively. Divergence between expected and relative survival became more evident at 3 years than at 1 year. Advanced age, male sex, and worse pre-fracture functional or cognitive status were associated with poorer survival in both cohorts. **Conclusions:** A second contralateral hip fracture may represent a clinically relevant marker of increased vulnerability and greater clinical complexity, associated with substantial excess mortality compared with general population matched by age and sex. Relative survival highlights the true prognostic burden of a primary or second fracture and supports intensive secondary prevention and tailored follow-up.

## 1. Introduction

Fragility hip fractures (FHF) represent one of the most serious adverse events affecting older adults, leading to substantial mortality, functional decline, long-term disability, and imposing a considerable burden on patients, their families, and healthcare systems [1,2,3]. While survival after a primary FHF has been widely investigated, the long-term prognostic implications of sustaining a second contralateral FHF remain insufficiently understood [4,5].

Existing evidence on long-term mortality after FHF in older adults shows heterogeneity and methodological limitations. A systematic review by Downey et al. reported a mean 1-year mortality rate of 22% (SD 7.2%), with estimates ranging from 2.4% to 34.8%, indicating substantial variability [6]. Other studies report mortality rates of approximately 30–33% at two years [7,8]. Rosinés et al. [9] reported overall survival rates of 37.7% (95% CI 35.6–39.9) at 5 years and 11.9% (95% CI 9.6–14.8) at 10 years. In that study, the cumulative probability of death following a hip fracture was 35.4% and 49.4% at 5 and 10 years respectively.

Patients who sustain a fragility fracture are at increased risk of experiencing a second fracture, regardless of the location of the initial event (i.e., vertebral, hip, or other sites). This risk is particularly high when the first fracture occurs at the hip, an event that is a strong predictor of subsequent contralateral fracture. Individuals with a prior hip fracture have a 3.7-fold higher risk of sustaining another hip fracture compared with those without this precedent [10].

The incidence of a second contralateral FHF after an initial FHF ranges from 2% to 15% depending on the follow-up period (1 to 5 years) and cohort characteristics, with an estimated cumulative incidence of 13% at 10 years. Notably, up to 60% of second fractures occur within four years of the index event, a period considered critical for secondary prevention [7,8,11,12,13,14,15,16].

The period following a first hip fracture also represents a critical window for rehabilitation and fall-prevention strategies. Early mobilization, appropriate weight-bearing progression, functional recovery, continuity of care, and structured rehabilitation pathways are central components of orthogeriatric management and may influence mobility, independence, fall risk, and the probability of subsequent fractures. In this context, healthcare professionals’ education and adherence to evidence-based rehabilitation recommendations are relevant to ensure consistent implementation of multidisciplinary post-fracture care [17,18].

A second fracture has been associated with increased mortality, longer hospital stay, and higher rates of postoperative complications [11,15,16]. Mortality may reach 24% at one year and 41.8% at five years [11,19,20,21]. However, some studies have reported divergent findings, raising doubts as to whether a second fracture truly entails significant excess mortality [22,23].

Traditional survival analyses in hip fracture research are often limited by incomplete or unreliable cause-of-death information, especially in frail older adults. This allows estimation of overall survival, but interpretation of relative survival remains limited when cause-of-death data are incomplete. The WebSurvCa application implements this framework using official mortality tables from the selected Spanish region. Relative survival was defined as the ratio between the observed survival of the study cohort and the expected survival of a comparable general population matched by age, sex, and calendar year. It provides an estimate of excess mortality associated with hip fracture without requiring individual cause-of-death information. Despite its methodological advantages, relative survival has been rarely applied in orthogeriatric research and has never been used to evaluate the impact after a second contralateral hip fracture.

Accordingly, the main aim of this study was to estimate overall survival and relative survival at 1 and 3 years in adults aged >65 years who underwent surgery for a first or second contralateral hip fracture. Secondary objectives were to explore survival patterns according to age, sex, functional dependence, and cognitive impairment.

## 2. Methods

### 2.1. Study Design and Ethical Approval

We conducted a retrospective hospital-based cohort study including all consecutive patients aged ≥65 years who underwent surgery for either a first hip fracture or a second contralateral hip fracture between 1 June 2010 and 31 December 2021. Ethical approval was obtained from the Independent Ethics Committee of the *Fundació Unió Catalana d’Hospitals* (CEIC 23/07, 15 February 2023). Informed consent was waived because of the retrospective design. The study complies with the Declaration of Helsinki and applicable national and institutional regulations.

The manuscript was prepared in accordance with the Strengthening the Reporting of Observational Studies in Epidemiology (STROBE) reporting guideline for observational cohort studies. A completed STROBE checklist is provided as Supplementary Material File S1.

### 2.2. Setting

The study took place in a single acute-care hospital serving a catchment population of 283,622 residents from five Catalan counties (Bages, Berguedà, Cerdanya, Moianès and Solsonès). During the study period, this hospital was the sole provider of acute surgical care for patients with hip fractures within the reference area. The Department of Orthopaedic Surgery and Traumatology records an annual mean of 3130 discharges, 44,692 outpatient consultations, 4081 surgeries, and 11,901 emergency department visits. Hip fractures in adults aged ≥65 years represent approximately 35% of surgical emergencies.

### 2.3. Study Subjects

Eligible patients are those aged ≥65 years who underwent surgery for either a first hip fracture or a second contralateral hip fracture. Exclusion criteria were pathological, high-energy, and periprosthetic fractures. Patients were classified into two cohorts according to fracture history: first hip fracture and second contralateral hip fracture. For patients with a second contralateral hip fracture, the second fracture episode was considered the index event for the present study, whereas the first fracture was used solely to establish fracture history. Because both fracture episodes were recorded in the hospital discharge database (UF3), previous contralateral hip fractures could be reliably identified. Patients in the second-fracture cohort constituted a conditional survivor group, because by definition they had survived the first fracture long enough to sustain a second event.

### 2.4. Study Period

Patients aged ≥65 years who underwent surgery for a first or second contralateral hip fracture between 1 June 2010 and 31 December 2021 were included. Follow-up extended from the date of surgery to the date of death or was censored on 31 December 2021, whichever occurred first.

### 2.5. Procedure

Data were collected from the Femur Fracture Functional Unit (UF3) prospective database. UF3 is a multidisciplinary unit established in 2010 and dedicated to the integrated care of adults ≥65 years of age undergoing surgery for a hip fracture. The UF3 team includes orthopaedic surgeons, internists, anesthesiologists, case management nurses, pharmacists, rheumatologists, rehabilitation specialists, social workers, and dietitians. Standardised perioperative protocols are used to reduce surgical delays, postoperative complications, hospital stay, readmissions at 30 days, and to improve survival outcomes. Postoperative rehabilitation is integrated into the UF3 multidisciplinary care pathway. According to the UF3 protocol, patients are encouraged to sit out of bed within 24–48 h after surgery and to start walking within 48–72 h, whenever their baseline clinical condition allowed it. Immediate weight-bearing was recommended in all patients. During hospital admission, an occupational therapist assesses the patient and family and provided education on activities of daily living and fall-prevention strategies. After discharge, patients continue home-based rehabilitation for approximately 1–2 months, according to clinical needs and expected benefit [24,25].

The UF3 database prospectively collects sociodemographic, perioperative and clinical information during hospital admission and includes structured follow-up information up to one year after surgery, including survival status, readmissions, complications, functional recovery, and living situation. One-year clinical follow-up variables other than survival were not included in the present analysis. Mortality status and date of death were obtained from the Spanish National Death Index.

### 2.6. Main Outcome

The primary outcomes were overall survival (OS), relative survival (RS), and the model-based probability of excess mortality associated with hip fracture (PHF) or from other causes (POC) at 1 and 3 years after surgery in patients with a first hip fracture and those with a second contralateral hip fracture.

### 2.7. Secondary Outcomes

Secondary outcomes were overall survival, relative survival, and the probability of dying due to hip fracture or from other causes at 1 and 3 years after surgery according to age, sex, functional dependence, and cognitive impairment.

### 2.8. Demographic and Clinical Measures

Process variables (admission date, surgery date, and discharge date) and sociodemographic variables (age, sex, living situation) were collected. Functional status for basic activities of daily living was assessed using the Barthel Index [26]. The degree of dependence was defined according to Shah et al.: i.e., 0–20 total, 21–60 severe, 61–90 moderate, 91–99 slight, and 100 independence [27]. Cognitive status was assessed using the Short Portable Mental Status Questionnaire (SPMSQ), classifying patients into four groups according to the number of errors: normal cognitive functioning (0–2), mild impairment (3–4), moderate impairment (5–7), and severe impairment (8–10) [28]. Baseline pre-fracture functional and cognitive status referred to the period before the index hip fracture. These data were recorded during admission by the UF3 team through clinical assessment and structured information obtained from the patient whenever possible, and from relatives, caregivers, or nursing home staff when appropriate. As indices of prior health status, the Charlson Comorbidity Index and the American Society of Anesthesiologists Physical Status Classification (ASA) were used [29,30]. The number of chronic medications (polypharmacy > 4 drugs) was recorded. Type of fracture and surgical delay were also assessed.

### 2.9. Statistical Analysis

Categorical variables are presented as absolute values and relative frequencies. Continuous variables are summarized as means and standard deviation for normal distribution and by the median and interquartile range (IQR) (25th to 75th percentiles) for non-normal distributions.

In the bivariate analysis, we used Student’s *t*-test or the non-parametric Mann–Whitney *U* test for continuous variables. We used the *χ*^2^ test for categorical variables, and Fisher’s exact test or bilateral exact *p*-values in contingency tables when the expected frequencies were less than five.

To determine overall survival (OS), relative survival (RS), the probability of dying from hip fracture (PHF), and the probability of dying from other causes (POC), the WebSurvCa application (https://shiny.snpstats.net/WebSurvCa/) was used (accessed in September 2025) [31]. OS represents the probability of survival after a given time since hip fracture, regardless of the cause of death. It is influenced by both mortality due to hip fracture and mortality due to other causes. The OS period was calculated as the difference between the date of surgery and the date of death from any cause or the final date of follow-up, which for patients alive was 31 December 2021. RS provides an estimate of excess mortality associated with hip fracture, without requiring individual cause-of-death information. RS has been used as a measure of the temporal evolution of the excess risk of death, considering the mortality of a reference population. It is calculated by dividing OS by the expected survival (ES) of the cohort based on the mortality of the general population (reference) to which they belong, matched by age, sex and calendar year. For the calculation of ES, the mortality rate of the population of the Spanish autonomous community of Catalonia was considered as the reference. From the ES, the excess risk of dying or excess mortality (EM) in the cohort is determined using the Ederer II method [32]. EM compares the observed mortality in our cohort to the expected mortality rate in the population of the autonomous community of Catalonia (Spain), adjusting for age distribution. The results can indicate how much higher or lower mortality is in our cohort compared to what would be expected in the general population. The EM measure helps to quantify the impact of hip fracture on mortality relative to the expected rates in the general population. Once the excess risk of death has been estimated, three probabilities can be computed at time T: (1) the crude PHF, (2) the crude POC, and (3) the probability of OS in the cohort.

The level of statistical significance was set at 5% (*p* < 0.05). The IBM SPSS Statistics v.29 (IBM Corporation^®^, Armonk, NY, USA) program and WebSurvCa application were used for statistical analysis.

Given the descriptive objective of the study and the limited number of patients in the second-fracture subgroup, no multivariable survival model was fitted; results are therefore presented as descriptive and stratified estimates.

## 3. Results

### 3.1. Baseline Characteristics of the Study Population

A total of 2467 patients with a primary FHF and 175 patients with a second contralateral fracture were included. Sociodemographic and clinical characteristics for both cohorts are presented in Table 1. Patients with a second fracture were significantly older, with a median age of 88.0 years (IQR: 83.7–91.1) compared with 86.4 years (IQR: 81.7–90.5) (*p* = 0.010), and included a lower proportion of patients in the 65–74-year range (2.9% vs. 9.2%; *p* = 0.014). A lower proportion of men (17.7% vs. 25.6%; *p* = 0.020) was observed in patients with a second fracture. The contralateral fracture cohort showed higher rates of institutionalization, comorbidity, functional dependence, and cognitive impairment. With respect to polypharmacy, a higher proportion of patients in the second fracture cohort were taking more than four medications (79.9% vs. 71.9%; *p* = 0.047). No significant differences were observed in anaesthetic risk according to ASA classification, type of fracture, surgical delay, or length of hospital stay.

### 3.2. Overall and Relative Survival at 1 and 3 Years

Table 2 and Table 3 summarize overall survival, relative survival, and the probability of dying due to hip fracture or from other causes at 1 and 3 years. At 1 year, survival estimates were broadly similar in both cohorts. At 3 years, the separation between observed and expected survival became more evident in both cohorts, and was greater after a second contralateral fracture. After a first fracture, 3-year overall survival was 53.8% (95% CI: 51.7–55.9) and relative survival was 70.5% (95% CI: 67.7–73.3). After a second contralateral fracture, the corresponding estimates were 43.7% (95% CI: 36.3–52.4) and 59.0% (95% CI: 49.1–70.8), respectively.

### 3.3. Survival Estimates According to Sex and Age Group

With regard to sex, both OS and RS were higher in women throughout the follow-up period in both cohorts. Among men with a primary fracture, the 1-year OS was 62.8% (95% CI: 59.1–66.7), and RS was 69.8% (95% CI: 65.6–74.1), whereas ES was 90.0%. In those with a contralateral fracture, OS was 53.9% (95% CI: 39.0–74.3), RS was 59.5% (95% CI: 43.1–82.1), whereas ES was 90.6%. PHF increased from 30.2% to 40.5% in men with a contralateral fracture. At 3 years, in men with a primary fracture, OS was 41.4% (95% CI: 37.5–45.8), RS was 57.3% (95% CI: 51.9–63.3), whereas ES was 72.3%. In those with a second contralateral fracture, OS was 26.9% (95% CI: 15.3–47.5), RS was 37.4% (95% CI: 21.2–65.9), whereas ES was 71.9%. PHF was 40.9% in patients with a primary fracture and 58.6% in those with a second fracture. Among women, the impact of a second fracture on 3-year OS and RS was less pronounced than in men.

As for age, a progressive decrease in survival with increasing age was observed in both cohorts. At 1 year of follow-up, the largest difference compared with ES was observed in patients aged 85 years or older in both cohorts. For patients older than 85 years with a primary fracture, 1-year OS was 68.4% (95% CI: 66.0–70.8), RS 77.8% (95% CI: 75.1–80.6), whereas ES was 87.9. In the cohort with a second contralateral hip fracture, 1-year OS was 69.0% (95% CI: 60.8–78.1), RS 77.9% (95% CI: 68.8–88.3), whereas ES was 88.6%.

PHF was 30.4% in patients with a primary fracture and 42.0% in those with a second fracture at 3 years. PHF was higher than POC across all age groups throughout follow-up.

### 3.4. Survival Estimates According to Cognitive and Functional Status

The poorer the cognitive status, the lower the OS and RS in both cohorts at 1 and 3 years. Relative to ES, the impact was progressively more pronounced in patients with mild, moderate, or severe impairment compared with those with normal functioning, at both 1 and 3 years of follow-up in both cohorts. PHF increased progressively with the degree of cognitive impairment. Among patients with mild, moderate, or severe impairment, PHF exceeded POC at both 1 and 3 years of follow-up. Conversely, in individuals with normal functioning, PHF was lower than POC at 3 years in both cohorts.

Regarding functional status, more independent individuals showed higher OS and RS in both cohorts at 1 and 3 years. In the primary-fracture cohort, compared with ES, differences were evident in patients with moderate, severe, and total dependence at both 1 and 3 years. In the contralateral-fracture cohort, compared with ES, differences were evident in patients with mild, moderate, severe, and total dependence at 3 years.

OS, RS, PHF and POC are presented in the Supplementary Data S1–S15 for each of the four years of follow-up, overall and according to sex, age, cognitive and functional status.

## 4. Discussion

This study provides descriptive estimates of overall and relative survival at 1 and 3 years in adults aged ≥65 years with a first or second contralateral hip fracture. Both cohorts showed similar patterns at 1 year, whereas differences between observed and expected survival became more evident at 3 years. Patients with a second contralateral fracture consistently showed poorer long-term overall and relative survival, particularly men, older adults, and those with functional or cognitive impairment. The probability of dying from a hip fracture was higher than the probability of dying from other causes in most subgroups; only adults with normal cognitive status were more likely to die from other causes.

At 3 years, individuals with a second contralateral fracture consistently showed lower overall and relative survival, both overall and when stratified by sex and age. These findings should be interpreted as associations rather than evidence of a causal or independent effect of the second contralateral fracture on survival. Patients with a second contralateral fracture were older and had poorer pre-fracture functional and cognitive status, higher comorbidity burden, higher institutionalization rates, and more frequent polypharmacy, all of which may have contributed to the observed survival differences. Accordingly, a second fracture may indicate a more advanced stage of clinical vulnerability rather than simply a repeated traumatic event.

This aligns with the hypothesis that the second fracture occurs at a point of critical decline in functional and physiological reserve. A second fracture may therefore indicate increased clinical vulnerability and greater prognostic complexity, rather than acting as an isolated determinant of survival, and RS appears to be a suitable metric for quantifying this accumulated risk [33].

Our results are consistent with previous literature reporting reduced survival after a second hip fracture.

Sobolev et al. reported a 55% increase in the mortality risk after a second hip fracture, regardless of sex or age [2], and Sağlam et al., documented five-year mortality rates as high as 66.5% [34]. The present study expands on prior evidence by providing stratified estimates and identifying subgroups at highest risk.

Differences are particularly relevant in men, who display a more pronounced decline in RS. This pattern has been described in other studies and may be related to a higher burden of comorbidity, and poorer recovery trajectories after hip fracture [35,36].

With respect to age, advanced age is a recognised prognostic factor for poorer survival. However, in our study, individuals aged 75–85, although not the oldest cohort, showed a marked reduction in long-term survival after a second fracture. This age range may represent a threshold at which physiological reserve becomes insufficient to compensate for the impact of a second major injury. Consequently, this group may benefit particularly from intensified secondary prevention and tailored rehabilitation strategies. In patients aged >85 years, survival decreased even further, consistent with the progressive decline in physiological resilience typically associated with advanced age.

We found that both functional and mental impairment play a key role in survival. Our data indicate that patients with severely impaired functional status or with cognitive decline prior to the fracture have a higher probability of dying in the following years in both cohorts. Pre-fracture functional status is a crucial factor for post-fracture survival and is clearly worsened by a second injury in patients with mild dependence. Patients with mild dependence (Barthel index 91–99) show a notable increase in PHF (from 8.4% to 20.6%) after a second fracture. This finding highlights that this group, often considered “functionally preserved”, may actually conceal subclinical frailty, and underscores the importance of functional assessment prior to fracture. These patients may benefit substantially from intensive rehabilitation programs and home support.

The degree of cognitive impairment is a strong predictor of mortality. Patients with severe impairment show a PHF of 63.6% at 3 years after a second fracture, with an OS of only 22.2%. The expected survival for this cohort in the general population is 70.7%. The second fracture increases this risk, indicating that cognitive frailty is strongly associated with increased clinical vulnerability. Lack of autonomy and difficulty actively engaging in recovery processes probably worsen the prognosis. Berry et al. (2007) and Dubljanin Raspopovic et al. (2020) reported that pre-fracture cognitive deficit is associated with more complications, poorer functional recovery, and increased risk of death [19,22].

In summary, a second FHF may be interpreted as a marker associated with increased vulnerability, greater clinical complexity, and poorer long-term survival. However, because no multivariable analyses were performed, our findings do not allow us to conclude that a second FHF is an independent prognostic factor for mortality. Our results highlight the importance of secondary prevention and the need to identify, and intervene in, high-risk patient groups in order to improve clinical outcomes and reduce the healthcare burden associated with these fractures. This study underlines the need to consider a second fracture as a warning indicator of increased clinical vulnerability, supporting the clinical importance of structured follow-up, fall-prevention measures, rehabilitation planning, and secondary fracture prevention after a primary fracture.

These components are clinically relevant because incomplete functional recovery, persistent mobility impairment, and recurrent falls may contribute to subsequent fracture risk. They are also consistent with orthogeriatric recommendations emphasizing early mobilization, multidisciplinary co-management, functional recovery, and continuity of care [18]. In addition, professional education and adherence to evidence-based rehabilitation recommendations are essential to ensure that these protocols are applied consistently in clinical practice [17]. However, the present study did not evaluate the effect of specific rehabilitation components on second contralateral fracture occurrence or survival.

## 5. Strengths and Limitations

One of the main strengths of this study is that, despite its retrospective cohort design, the information was obtained from a prospective database that has systematically collected data from admission until one-year post-intervention since 2010. In addition, the inclusion of pre-fracture functional and cognitive measures allowed a clinically meaningful stratified description of prognosis in relevant vulnerable subgroups.

Another strength is the use of a novel data analysis approach, previously applied in oncology, which provides a new perspective on survival in patients with a primary or secondary contralateral hip fracture in comparison with the general population. This innovative approach provided an additional perspective of the likelihood of survival in these patients. Relative survival estimated with the WebSurvCa application provides survival due to hip fracture without requiring adjudicated cause-of-death data which are often incomplete or unreliable in older adults with multimorbidity. Relative survival provides more accurate information than overall survival and better characterizes the long-term prognostic burden associated with first and second hip fractures. By using RS as the primary outcome, this study bridges the gap between clinical observation and population-based expectations. This approach allows clinicians to interpret survival after hip fracture not only in absolute terms but in the context of the expected life span for individuals of similar age and sex in the general population.

The main limitation of the study is its single-centre design, which may limit external validity. However, assuming that all hip fractures require hospitalization and that our hospital is the only one in our geographical area, the UF3’s coverage can be considered almost complete. Since its creation, the UF3 has consistently maintained the same operational methods and care protocols. Despite the robustness of the dataset, a methodological limitation is the relatively small sample size of patients with a second contralateral FHF (*n* = 175, 6.6%) compared with those with a primary fracture (*n* = 2467). This asymmetry may have reduced the precision of the 1- and 3-year estimates in the cohort of patients with contralateral fractures particularly in subgroup analyses stratified by sex, age, cognitive status, and functional status. Accordingly, these subgroup findings should be interpreted with caution and should be considered exploratory and hypothesis-generating. This limitation does not invalidate the results but calls for caution in their generalization and underlines the need for future multicentre studies with larger samples to confirm and expand these findings. In addition, we did not include a validated measure of frailty. Instead, functional dependence, cognitive impairment, comorbidity, ASA class, and polypharmacy were used as clinical proxies of vulnerability; however, residual confounding related to frailty cannot be excluded.

Frailty was not directly assessed using a validated frailty instrument. Therefore, functional dependence, cognitive impairment, comorbidity, ASA classification, and polypharmacy were used as surrogate measures of vulnerability rather than as direct measures of frailty. Consequently, our findings do not allow definitive conclusions regarding frailty progression. Future studies should incorporate validated frailty assessment tools to better characterize frailty and to determine its independent prognostic contribution to survival after a primary or second contralateral hip fracture.

Another important limitation is the lack of systematic information on osteoporosis treatment and secondary fracture prevention during most of the study period. At our institution, a structured fracture liaison service and a protocolized anti-osteoporotic treatment pathway after hip fracture were introduced from 2022 onwards. Therefore, during most of the study period, FLS-based secondary prevention and systematic osteoporosis treatment were not yet implemented, and these data were not available in a complete and standardized manner. Consequently, we could not assess whether differences in osteoporosis management, treatment adherence, or fall-prevention strategies contributed to the occurrence of second contralateral fractures or to subsequent survival.

Although a standardized rehabilitation pathway was part of the UF3 care model, the UF3 database does not include individual-level information on adherence to mobilization targets, rehabilitation intensity, functional progress during home-based rehabilitation, or continuity of post-discharge rehabilitation. Therefore, the potential contribution of these factors to survival could not be assessed in the present study.

Finally, the second-fracture cohort is subject to survivor-selection bias, because patients had to survive the first fracture and remain at risk long enough to sustain a second contralateral fracture. Therefore, comparisons between first and second fractures should be interpreted as descriptive and prognostic rather than causal.

## 6. Conclusions

Adults aged ≥65 years with a second contralateral hip fracture showed poorer 3-year overall and relative survival than those with a first hip fracture. These differences were particularly evident at 3 years than at 1 year, in men, older adults, and patients with worse pre-fracture functional or cognitive status.

A second contralateral hip fracture should therefore be interpreted as a warning indicator of increased clinical vulnerability and greater prognostic complexity. These findings may help clinicians identify patients who require closer follow-up, comprehensive geriatric assessment, rehabilitation planning, and careful consideration of secondary fracture prevention, although specific osteoporosis treatment and fall-prevention interventions were not assessed in this study.

## Figures and Tables

**Table 1:** Baseline characteristics. Overall and by primary versus second contralateral hip fracture.

	Primary Femur Fracture	Contralateral Hip Fracture	*p*-Value
	*n* = 2467	*n* = 175	
Age (years)	86.4 [81.7–90.5]	88.0 [83.7–91.1]	0.010
65–74	227 (9.2%)	5 (2.9%)	0.014
75–85	807 (32.7%)	58 (33.1%)	
>85	1433 (58.1%)	112 (64.0%)	
Gender			0.020
Men	631 (25.6%)	31 (17.7%)	
Women	1836 (74.4%)	144 (82.3%)	
Living situation			0.003
Alone	463 (18.8%)	26 (14.9%)	
Family	1438 (58.3%)	89 (50.9%)	
Nursing home	566 (22.9%)	60 (34.3%)	
Number of drugs	6 [4–9]	7 [5–10]	0.017
≤4	693 (28.1%)	37 (21.1%)	0.047
>4	1774 (71.9%)	138 (79.9%)	
ASA			0.126
I	14 (0.6%)	0 (0.0%)	
II	818 (33.2%)	45 (25.7%)	
III	1491 (60.4%)	117 (66.9%)	
IV	144 (5.8%)	13 (7.4%)	
Functional status (Barthel index)	80 [60–95]	65 [50–80]	<0.001
Independence (100)	496 (20.1%)	3 (1.7%)	<0.001
Mild dependence (91–99)	195 (7.9%)	9 (5.1%)	
Moderate dependence (61–90)	1078 (43.7%)	82 (46.9%)	
Severe dependence (21–60)	616 (25.0%)	70 (40.0%)	
Total dependence (0–20)	82 (3.3%)	11 (6.3%)	
Cognitive status (SPMSQ)	3 [0–7]	5 [1–8]	<0.001
Normal functioning (0–2)	1191 (48.3%)	60 (34.3%)	0.004
Mild impairment (3–4)	303 (12.3%)	24 (13.7%)	
Moderate impairment (5–7)	377 (15.3%)	35 (20.0%)	
Severe impairment (8–10)	596 (24.2%)	56 (32.0%)	
Charlson comorbidity index	2 [1–3]	2 [1–4]	0.012
Absence of comorbidity (0–1)	969 (39.3%)	58 (33.1%)	0.040
Low comorbidity (2)	512 (20.8%)	30 (17.1%)	
High comorbidity (3 or more)	986 (40.0%)	87 (49.7%)	
Fracture type			0.282
Femoral neck	542 (22.0%)	45 (25.7%)	
Intertrochanteric	1558 (63.2%)	100 (57.1%)	
Subtrochanteric	367 (14.9%)	30 (17.1%)	
Surgical delay (hours)	48 [24–72]	48 [24–72]	0.411
≤48	1580 (64.0%)	109 (62.3%)	0.639
>48	887 (36.0%)	66 (37.7%)	
Length of stay (days)	9 [7–12]	8 [6–11]	0.113

*n* (%); media^n^ [25th–75th percentile]. For variables presented as both continuous and categorical, separate *p* values are reported. The *p* value for the categorized variable refers to the overall comparison of category distributions between groups.

**Table 2:** One-year survival and probability of death due to hip fracture.

		Primary Femur Fracture						Contralateral Hip Fracture				
	ES	No. at Risk	OS	RS	PHF	POC	ES	No. at Risk	OS	RS	PHF	POC
**All**	91.7%	1651	74.8%	81.6%	18.4%	6.8%	91.3%	119	73.3%	80.3%	19.7%	7.0%
**Gender**												
Men	90.0%	356	62.8%	69.8%	30.2%	7.0%	90.6%	16	53.9%	59.5%	40.5%	5.6%
Women	92.3%	1296	79.0%	85.6%	14.4%	6.6%	91.4%	103	77.4%	84.7%	15.3%	7.3%
**Age (years)**												
65–74	98.6%	171	85.9%	87.1%	12.9%	1.2%	93.6%	4	80.0%	85.5%	19.6%	0.4%
75–85	96.0%	614	83.1%	86.6%	13.4%	3.5%	96.3%	42	81.0%	84.1%	15.9%	3.1%
>85	87.9%	867	68.4%	77.8%	22.2%	9.4%	88.6%	73	69.0%	77.9%	22.1%	8.9%
**Cognitive status (SPMSQ)**												
Normal functioning (0–2)	93.0%	921	84.9%	91.3%	8.7%	6.4%	92.3%	49	86.5%	93.7%	6.3%	7.2%
Mild impairment (3–4)	90.7%	208	73.3%	80.8%	19.2%	7.5%	91.5%	18	78.9%	86.2%	13.8%	7.3%
Moderate impairment (5–7)	90.1%	215	67.0%	74.4%	25.6%	7.4%	92.1%	25	73.8%	80.1%	19.9%	6.3%
Severe impairment (8–10)	90.2%	309	60.1%	66.6%	33.4%	6.5%	90.0%	27	55.8%	62.0%	38.0%	6.2%
**Functional status (Barthel index)**												
Independence (100)	94.7%	405	90.7%	95.8%	4.2%	5.1%	100%	2	100.0%	100.0%	0.0%	0.0%
Mild dependence (91–99)	92.6%	152	86.3%	93.2%	6.8%	6.9%	97.9%	8	77.8%	79.4%	20.6%	1.6%
Moderate dependence (61–90)	90.8%	727	74.7%	82.3%	17.7%	7.6%	92.2%	58	75.3%	81.7%	18.3%	6.4%
Severe dependence (21–60)	90.3%	325	60.8%	67.3%	32.7%	6.5%	90.3%	42	66.2%	73.3%	26.7%	7.1%
Total dependence (0–20)	89.6%	43	56.7%	63.3%	36.7%	6.6%	98.3%	10	90.9%	92.5%	7.5%	1.6%

ES: expected survival; No. at risk: number of individuals at risk at the start of the interval; OS: overall survival; RS: relative survival; PHF: model-based probability of excess mortality associated with hip fracture; POC: probability of dying from other causes.

**Table 3:** Three-year survival and probability of death due to hip fracture.

		Primary Femur Fracture						Contralateral Hip Fracture				
	ES	No. at Risk	OS	RS	PHF	POC	ES	No. at Risk	OS	RS	PHF	POC
**All**	76.3%	949	53.8%	70.5%	28.1%	18.1%	74.1%	49	43.7%	59.0%	38.0%	18.3%
**Gender**												
Men	72.3%	194	41.4%	57.3%	40.9%	17.7%	71.9%	8	26.9%	37.4%	58.6%	14.5%
Women	77.4%	756	58.0%	74.9%	23.8%	18.2%	74.5%	41	47.7%	64.0%	33.2%	19.1%
**Age (years)**												
65–74	95.1%	116	75.8%	79.7%	20.2%	4.0%	91.3%	2	40.0%	43.8%	59.0%	1.0%
75–85	87.4%	396	63.8%	73.0%	26.2%	10.0%	87%	19	56.7%	65.2%	33.6%	9.7%
>85	65.8%	438	44.5%	67.6%	30.4%	25.1%	65.6	26	35.1%	53.5%	42.0%	22.9%
**Cognitive status (SPMSQ)**												
Normal functioning (0–2)	79.1%	586	68.7%	86.9%	12.6%	18.7%	76.1%	23	61.0%	80.2%	17.9%	21.2%
Mild impairment (3–4)	73.4%	125	51.6%	70.3%	28.6%	19.8%	71.6%	9	56.7%	79.2%	22.0%	21.3%
Moderate impairment (5–7)	72.2%	98	40.5%	56.1%	41.1%	18.4%	75.4%	8	38.7%	51.3%	45.5%	15.8%
Severe impairment (8–10)	73.0%	143	32.4%	44.4%	52.5%	15.1%	70.7%	8	22.2%	31.4%	63.6%	14.2%
**Functional status (Barthel index)**												
Independence (100)	83.5%	299	77.5%	92.8%	6.9%	15.6%	0.0% *	1 *	0.0%	15.3%	84.7%	15.3%
Mild dependence (91–99)	77.6%	101	73.4%	94.6%	8.4%	18.2%	89.5%	6	77.8%	86.9%	20.6%	1.6%
Moderate dependence (61–90)	73.9%	387	52.1%	70.5%	28.0%	19.9%	75.7%	26	56.7%	74.9%	24.2%	19.1%
Severe dependence (21–60)	72.8%	146	34.3%	47.1%	50.0%	15.7%	71.6%	18	31.3%	43.7%	51.7%	17.0%
Total dependence (0–20)	67.6%	18	25.2%	37.3%	58.6%	16.2%	74.3%	1	13.6%	18.3%	75.4%	11.0%

ES: expected survival; No. at risk: number of individuals at risk at the start of the interval; OS: overall survival; RS: relative survival; PHF: model-based probability of excess mortality associated with hip fracture; POC: probability of dying from other causes. * Survival at 2 years.

## Data Availability

The data presented in this study are available on reasonable request from the corresponding author. The data are not publicly available due to privacy and ethical restrictions involving clinical patient information.

## Supplementary Materials

The following supporting information can be downloaded at: https://www.mdpi.com/article/10.3390/geriatrics11040083/s1, File S1: STROBE Checklist for Cohort Studies; Data S1: Long-term survival and probability of death due to hip fracture; Data S2: Long-term survival and probability of death due to hip fracture in male; Data S3: Long-term survival and probability of death due to hip fracture in female; Data S4: Long-term survival and probability of death due to hip fracture in patients aged 65 to 74 years; Data S5: Long-term survival and probability of death due to hip fracture in patients aged 75 to 84 years; Data S6: Long-term survival and probability of death due to hip fracture in patients 85 years of age and older; Data S7: Long-term survival and probability of death due to hip fracture in patients with normal cognitive functioning (0–2 errors in Short Portable Mental Status Questionnaire); Data S8: Long-term survival and probability of death due to hip fracture in patients with mild cognitive impairment (3–4 errors in Short Portable Mental Status Questionnaire); Data S9: Long-term survival and probability of death due to hip fracture in patients with moderate cognitive impairment (5–7 errors in Short Portable Mental Status Questionnaire); Data S10: Long-term survival and probability of death due to hip fracture in patients with severe cognitive impairment (8 or more errors in Short Portable Mental Status Questionnaire); Data S11: Long-term survival and probability of death due to hip fracture in patients with independence for basic Activities of Daily Living (score 100 in Barthel Index); Data S12: Long-term survival and probability of death due to hip fracture in patients with slight dependence for basic Activities of Daily Living (score 91–99 in Barthel Index); Data S13: Long-term survival and probability of death due to hip fracture in patients with moderate dependence for basic Activities of Daily Living (score 61–90 in Barthel Index); Data S14: Long-term survival and probability of death due to hip fracture in patients with severe dependence for basic Activities of Daily Living (score 21–60 in Barthel Index); Data S15: Long-term survival and probability of death due to hip fracture in patients with total dependence for basic Activities of Daily Living (score 0–20 in Barthel Index).
