# Supplementary material for: Overall and Relative Survival After a Second Contralateral Hip Fracture in Adults over 65 Years: A Retrospective Cohort Study
Source: Geriatrics (Basel). 2026 Jul 10;11(4):83. doi: 10.3390/geriatrics11040083 (PMC13398190; doi:10.3390/geriatrics11040083)
Supplement: Supplementary file 1 [file geriatrics-11-00083-s001.zip › geriatrics-4317069-supplementary.pdf]

Supplementary Materials

The following supporting information can be downloaded: Overall survival, relative survival, probability of death due to hip fracture, and probability of death due to other causes during the first four years of follow-up, overall and stratified by sex, age, cognitive status, and functional status.

Supplementary Material File S1: STROBE Checklist for Cohort Studies

Overall and Relative Survival After a Second Contralateral Hip Fracture in Adults over 65 Years: A Retrospective Cohort Study

Section/Topic	Item No.	STROBE recommendation	Reported on page/location in manuscript
Title and abstract	1a	Indicate the study's design with a commonly used term in the title or the abstract.	Title page and Abstract, pp. 1: "Retrospective Cohort Study" in the title and "Retrospective cohort study" in the Abstract.
Title and abstract	1b	Provide in the abstract an informative and balanced summary of what was done and what was found.	Abstract, pp. 1: background/objectives, methods, results, and conclusions summarize the cohort, survival methods, main OS/RS estimates, and interpretation.
Introduction – Background/rationale	2	Explain the scientific background and rationale for the investigation being reported.	Introduction, pp. 2–3: burden of fragility hip fracture, risk of second contralateral fracture, period after first fracture as a critical window for rehabilitation and fall prevention, limitations of conventional mortality analyses, and rationale for relative survival.
Introduction – Objectives	3	State specific objectives, including any prespecified hypotheses.	End of Introduction, p. 3: main aim to estimate 1- and 3-year OS and RS after first and second contralateral hip fracture; secondary objectives by sex, age, functional dependence, and cognitive impairment.
Methods – Study design	4	Present key elements of study design early in the paper.	Methods 2.1, p. 3: retrospective hospital-based cohort study of consecutive patients undergoing surgery for first or second contralateral hip fracture.
Methods – Setting	5	Describe the setting, locations, and relevant dates, including periods of recruitment, exposure, follow-up, and data collection.	Methods 2.2 and 2.4, pp. 3: single acute-care reference hospital, catchment population and counties, study period from 1 June 2010 to 31 December 2021, follow-up to death or censoring date.
Methods – Participants	6a	Cohort study: Give the eligibility criteria, and the sources and methods of selection of participants. Describe methods of follow-up.	Methods 2.3–2.5, pp. 3–4: eligible patients aged ≥65 years surgically treated for first or second contralateral hip fracture; exclusions; data from the UF3 prospective database; both fractures in the second-fracture cohort recorded within the UF3 system; mortality from the Spanish National Death Index; UF3 rehabilitation pathway and post-discharge home-based rehabilitation described.
Methods – Participants	6b	Cohort study: For matched studies, give matching criteria and number of exposed and unexposed.	Not applicable. This was not a matched cohort study.
Methods – Variables	7	Clearly define all outcomes, exposures, predictors, potential confounders, and effect modifiers. Give diagnostic criteria, if applicable.	Methods 2.6–2.8, pp. 4: primary and secondary survival outcomes; fracture cohort definition; demographic and clinical variables; Barthel Index, SPMSQ, Charlson, ASA, polypharmacy, fracture type, surgical delay, and pre-fracture functional/cognitive status. The UF3 postoperative rehabilitation pathway is described in Methods 2.5, but individual rehabilitation variables were not analysed as study outcomes or predictors.
Methods – Data sources/measurement	8	For each variable of interest, give sources of data and details of methods of assessment/measurement. Describe comparability of assessment methods if there is more than one group.	Methods 2.5 and 2.8, pp. 3: UF3 database, clinical assessment, structured information from patient/relatives/caregivers/nursing home staff for pre-fracture functional and cognitive status; mortality status and date of death from the Spanish National Death Index. Methods 2.5 also describes the UF3 rehabilitation pathway, including early sitting, early walking, immediate weight-bearing, occupational therapy, fall-prevention education, and home-based rehabilitation.

Section/Topic	Item No.	STROBE recommendation	Reported on page/location in manuscript
Methods – Bias	9	Describe any efforts to address potential sources of bias.	Methods 2.3 and 2.9, pp. 3–5; Limitations, pp. 11-12: consecutive cohort from the only regional acute surgical centre; explicit recognition of conditional survivor group, no causal inference, lack of multivariable modelling, small second-fracture subgroup, lack of validated frailty measure, lack of systematic osteoporosis/FLS data for most of the study period, and lack of individual-level rehabilitation variables.
Methods – Study size	10	Explain how the study size was arrived at.	Methods/Results, pp. 3: all consecutive eligible patients during the study period were included; final cohort reported as 2,642 patients. No formal sample size calculation was performed because this was a retrospective cohort including all eligible cases.
Methods – Quantitative variables	11	Explain how quantitative variables were handled in the analyses. If applicable, describe which groupings were chosen and why.	Methods 2.8–2.9, pp. 4–5: continuous variables summarized by mean/SD or median/IQR; Barthel, SPMSQ, Charlson, ASA, polypharmacy, surgical delay, sex and age group categories defined.
Methods – Statistical methods	12a	Describe all statistical methods, including those used to control for confounding.	Methods 2.9, pp. 4-5: bivariate comparisons, survival analyses using WebSurvCa, Ederer II method, significance threshold, SPSS and WebSurvCa. No multivariable model was fitted due to the descriptive objective and limited number of second-fracture patients.
Methods – Statistical methods	12b	Describe any methods used to examine subgroups and interactions.	Methods 2.7 and 2.9, pp. 4–5; Results 3.3–3.4, pp. 7: stratified estimates by age, sex, functional dependence, and cognitive impairment; presented as exploratory and hypothesis-generating.
Methods – Statistical methods	12c	Explain how missing data were addressed.	Methods 2.9, pp. 4-5: analyses were performed using the available data for each variable, and no imputation of missing values was conducted.
Methods – Statistical methods	12d	Cohort study: If applicable, explain how loss to follow-up was addressed.	Methods 2.4–2.5, p. 3: follow-up extended from surgery to death or censoring on 31 December 2021; mortality status and date of death obtained from Spanish National Death Index.
Methods – Statistical methods	12e	Describe any sensitivity analyses.	Not applicable. No sensitivity analyses were performed.
Results – Participants	13a	Report numbers of individuals at each stage of study: potentially eligible, examined for eligibility, confirmed eligible, included, completing follow-up, and analysed.	Results 3.1, p. 6: total included and analysed patients reported as 2,467 first hip fractures and 175 second contralateral hip fractures. Tables 2–3 report numbers at risk at 1 and 3 years.
Results – Participants	13b	Give reasons for non-participation at each stage.	Methods 2.3 and Results 3.1, pp. 3 and 5: exclusion criteria stated and final cohort reported. Detailed excluded counts are not reported because the cohort consisted of consecutive eligible surgical cases recorded in the UF3 database.
Results – Participants	13c	Consider use of a flow diagram.	A flow diagram was not included. Cohort counts are reported in Results and Tables 1–3.
Results – Descriptive data	14a	Give characteristics of study participants and information on exposures and potential confounders.	Results 3.1 and Table 1, pp. 5–6: sociodemographic, clinical, functional and cognitive characteristics by first versus second contralateral hip fracture.
Results – Descriptive data	14b	Indicate number of participants with missing data for each variable of interest.	Table 1 reports available data for the variables presented. Methods 2.9 states that analyses were performed using available data and no imputation was conducted.
Results – Descriptive data	14c	Cohort study: Summarise follow-up time, for example average and total amount.	Methods 2.4, p. 3: follow-up from surgery to death or censoring on 31 December 2021; Results/Tables 2–3 report 1- and 3-year survival estimates.

Section/Topic	Item No.	STROBE recommendation	Reported on page/location in manuscript
Results – Outcome data	15	Cohort study: Report numbers of outcome events or summary measures over time.	Results 3.2–3.5 and Tables 2–3, pp. 6–9: OS, RS, ES, PHF, POC, and numbers at risk at 1 and 3 years.
Results – Main results	16a	Give unadjusted estimates and, if applicable, confounder-adjusted estimates and their precision. Make clear which confounders were adjusted for and why.	Results 3.2–3.4 and Tables 2–3, pp. 7-9: descriptive, unadjusted survival estimates with available 95% CIs for OS and RS. No adjusted model was fitted, as stated in Methods.
Results – Main results	16b	Report category boundaries when continuous variables were categorized.	Methods 2.8 and Table 1, pp. 4 and 6: age groups, Barthel categories, SPMSQ categories, Charlson categories, polypharmacy threshold and surgical delay threshold are reported.
Results – Main results	16c	If relevant, consider translating estimates of relative risk into absolute risk for a meaningful time period.	Results 3.2 and Tables 2–3, pp. 7-9: absolute survival percentages and model-based probabilities at 1 and 3 years are reported, allowing clinically interpretable estimates.
Results – Other analyses	17	Report other analyses done, for example analyses of subgroups and interactions, and sensitivity analyses.	Results 3.3–3.4, pp. 7; Supplementary Material: stratified survival estimates by sex, age, cognitive status and functional status. No interaction or sensitivity analyses were performed. Rehabilitation components were discussed clinically but not analysed as subgroup or exposure variables.
Discussion – Key results	18	Summarise key results with reference to study objectives.	Discussion, pp. 10-11: summarizes 1- and 3-year OS/RS, poorer survival after second contralateral fracture, higher-risk subgroups, and clinical relevance of rehabilitation, fall prevention, and secondary prevention.
Discussion – Limitations	19	Discuss limitations of the study, taking into account sources of potential bias or imprecision.	Strengths and Limitations, pp. 11-12: single-centre design, small second-fracture subgroup, exploratory subgroup analyses, no validated frailty measure, no systematic osteoporosis/FLS data during most of the study period, survivor-selection bias, and lack of analysis of detailed individual rehabilitation variables such as adherence to mobilization targets, rehabilitation intensity, functional progress during home-based rehabilitation, and post-discharge rehabilitation continuity/adherence.
Discussion – Interpretation	20	Give a cautious overall interpretation of results considering objectives, limitations, multiplicity of analyses, results from similar studies, and other relevant evidence.	Discussion and Conclusions, pp. 10-12: results are interpreted as associations, not causal effects; RS interpreted as excess mortality rather than cause-specific mortality; findings compared with previous literature; rehabilitation and secondary prevention are discussed as clinically relevant but not directly evaluated interventions.
Discussion – Generalisability	21	Discuss the generalisability/external validity of the study results.	Strengths and Limitations, p. 11-12: single-centre design may limit external validity, but the hospital is the only acute surgical centre for hip fractures in the reference area, supporting near-complete regional coverage.
Other information – Funding	22	Give the source of funding and the role of the funders for the present study and, if applicable, for the original study on which the present article is based.	Funding statement, p. 13: “This research received no external funding.”

Checklist based on the STROBE Statement checklist for cohort studies.

Supplementary Data S1. Long-term survival and probability of death due to hip fracture.

PRIMARY FEMUR FRACTURE

T	ES	No. at risk	OS	95%CI OS		RS	95%CI RS		SRI	PHF	POC
**0**	1.000	2467	1	1	1	1	1	1	1	0	0
**1**	0.917	1651	0.748	0.731	0.766	0.816	0.797	0.835	0.816	0.184	0.068
**2**	0.838	1275	0.638	0.619	0.658	0.761	0.738	0.785	0.933	0.234	0.128
**3**	0.763	949	0.538	0.517	0.559	0.705	0.677	0.733	0.926	0.281	0.181
**4**	0.691	665	0.433	0.412	0.456	0.627	0.596	0.660	0.890	0.341	0.227

CONTRALATERAL FEMUR FRACTURE

T	ES	No. at risk	OS	95%CI OS		RS	95%CI RS		SRI	PHF	POC
**0**	1.000	175	1	1	1	1	1	1	1	0	0
**1**	0.913	119	0.733	0.670	0.802	0.803	0.734	0.879	0.803	0.197	0.070
**2**	0.824	88	0.601	0.531	0.681	0.729	0.643	0.826	0.907	0.265	0.134
**3**	0.741	49	0.437	0.363	0.524	0.590	0.491	0.708	0.809	0.380	0.183
**4**	0.664	33	0.355	0.281	0.448	0.535	0.425	0.675	0.908	0.420	0.225

T: annual interval, survival in the corresponding year. 0: start of study; 1 year: survival up to that year; etc.; ES: expected survival; No at risk: number of individuals at risk at the at the start of the interval; OS: overall survival; 95%CI OS: lower and upper limit of the 95% confidence interval of overall survival; RS: relative survival; 95%CI RS: lower and upper limit of the 95% confidence interval of relative survival; SRI: relative survival in the annual interval; PHF: Probability of dying from hip fracture; POC: Probability of dying from other causes.

Supplementary Data S2. Long-term survival and probability of death due to hip fracture in male.

PRIMARY FEMUR FRACTURE

T	ES	No. at risk	OS	95%CI OS		RS	95%CI RS		SRI	PHF	POC
**0**	1.000	631	1	1	1	1	1	1	1	0	0
**1**	0.900	356	0.628	0.591	0.667	0.698	0.656	0.741	0.698	0.302	0.070
**2**	0.807	256	0.505	0.466	0.547	0.626	0.577	0.678	0.897	0.367	0.128
**3**	0.723	194	0.414	0.375	0.458	0.573	0.519	0.633	0.916	0.409	0.177
**4**	0.641	132	0.329	0.291	0.373	0.513	0.453	0.581	0.894	0.453	0.218

CONTRALATERAL FEMUR FRACTURE

T	ES	No. at risk	OS	95%CI OS		RS	95%CI RS		SRI	PHF	POC
**0**	1.000	31	1	1	1	1	1	1	1	0	0
**1**	0.906	16	0.539	0.390	0.743	0.595	0.431	0.821	0.595	0.405	0.056
**2**	0.812	14	0.471	0.326	0.681	0.580	0.401	0.838	0.974	0.419	0.110
**3**	0.719	8	0.269	0.153	0.475	0.374	0.212	0.659	0.645	0.586	0.145
**4**	0.618	4	0.157	0.070	0.350	0.254	0.114	0.565	0.678	0.673	0.170

T: annual interval, survival in the corresponding year. 0: start of study; 1 year: survival up to that year; etc.; ES: expected survival; No at risk: number of individuals at risk at the at the start of the interval; OS: overall survival; 95%CI OS: lower and upper limit of the 95% confidence interval of overall survival; RS: relative survival; 95%CI RS: lower and upper limit of the 95% confidence interval of relative survival; SRI: relative survival in the annual interval; PHF: Probability of dying from hip fracture; POC: Probability of dying from other causes.

Supplementary Data S3. Long-term survival and probability of death due to hip fracture in female.

PRIMARY FEMUR FRACTURE

T	ES	No. at risk	OS	95%CI OS		RS	95%CI RS		SRI	PHF	POC
**0**	1.000	1836	1	1	1	1	1	1	1	0	0
**1**	0.923	1296	0.790	0.771	0.809	0.856	0.836	0.877	0.856	0.144	0.066
**2**	0.848	1020	0.684	0.662	0.707	0.807	0.781	0.834	0.943	0.189	0.127
**3**	0.774	756	0.580	0.556	0.605	0.749	0.718	0.781	0.928	0.238	0.182
**4**	0.704	534	0.469	0.444	0.496	0.666	0.630	0.703	0.889	0.303	0.228

CONTRALATERAL FEMUR FRACTURE

T	ES	No. at risk	OS	95%CI OS		RS	95%CI RS		SRI	PHF	POC
**0**	1.000	144	1	1	1	1	1	1	1	0	0
**1**	0.914	103	0.774	0.709	0.846	0.847	0.775	0.925	0.847	0.153	0.073
**2**	0.829	74	0.629	0.552	0.717	0.759	0.667	0.865	0.897	0.233	0.138
**3**	0.745	41	0.477	0.395	0.576	0.640	0.530	0.772	0.842	0.332	0.191
**4**	0.672	29	0.410	0.326	0.516	0.610	0.485	0.768	0.954	0.354	0.236

T: annual interval, survival in the corresponding year. 0: start of study; 1 year: survival up to that year; etc.; ES: expected survival; No at risk: number of individuals at risk at the at the start of the interval; OS: overall survival; 95%CI OS: lower and upper limit of the 95% confidence interval of overall survival; RS: relative survival; 95%CI RS: lower and upper limit of the 95% confidence interval of relative survival; SRI: relative survival in the annual interval; PHF: Probability of dying from hip fracture; POC: Probability of dying from other causes.

Supplementary Data S4. Long-term survival and probability of death due to hip fracture in patients aged 65 to 74 years.

PRIMARY FEMUR FRACTURE

T	ES	No. at risk	OS	95%CI OS		RS	95%CI RS		SRI	PHF	POC
**0**	1.000	227	1	1	1	1	1	1	1	1	1
**1**	0.986	171	0.859	0.814	0.906	0.871	0.825	0.919	0.871	0.129	0.012
**2**	0.968	142	0.795	0.742	0.852	0.821	0.766	0.879	0.942	0.179	0.026
**3**	0.951	116	0.758	0.701	0.820	0.797	0.737	0.862	0.971	0.202	0.040
**4**	0.933	89	0.687	0.622	0.759	0.736	0.667	0.813	0.924	0.260	0.054

CONTRALATERAL FEMUR FRACTURE

T	ES	No. at risk	OS	95%CI OS		RS	95%CI RS		SRI	PHF	POC
**0**	0.995	5	1	0	1	1	0	1	1	0	0
**1**	0.936	4	0.800	0.408	1.000	0.855	0.509	1.000	1.000	0.196	0.004
**2**	0.931	3	0.800	0.347	1.000	0.860	0.467	1.000	1.000	0.196	0.004
**3**	0.913	2	0.400	0.000	1.000	0.438	0.000	1.000	0.510	0.590	0.010
**4**	0.000	1	0.000	0.000	0.000	0.032	0.000	0.378	0.073	0.970	0.030

T: annual interval, survival in the corresponding year. 0: start of study; 1 year: survival up to that year; etc.; ES: expected survival; No at risk: number of individuals at risk at the at the start of the interval; OS: overall survival; 95%CI OS: lower and upper limit of the 95% confidence interval of overall survival; RS: relative survival; 95%CI RS: lower and upper limit of the 95% confidence interval of relative survival; SRI: relative survival in the annual interval; PHF: Probability of dying from hip fracture; POC: Probability of dying from other causes.

Supplementary Data S5. Long-term survival and probability of death due to hip fracture in patients aged 75 to 84 years.

PRIMARY FEMUR FRACTURE

T	ES	No. at risk	OS	95%CI OS		RS	95%CI RS		SRI	PHF	POC
**0**	1.000	807	1	1	1	1	1	1	1	0	0
**1**	0.960	614	0.831	0.806	0.858	0.866	0.840	0.894	0.866	0.134	0.035
**2**	0.918	492	0.723	0.691	0.756	0.788	0.754	0.823	0.909	0.210	0.067
**3**	0.874	396	0.638	0.603	0.674	0.730	0.691	0.772	0.927	0.262	0.100
**4**	0.829	309	0.563	0.526	0.601	0.679	0.635	0.725	0.930	0.307	0.130

CONTRALATERAL FEMUR FRACTURE

T	ES	No. at risk	OS	95%CI OS		RS	95%CI RS		SRI	PHF	POC
**0**	1.000	58	1	1	1	1	1	1	1	0	0
**1**	0.963	42	0.810	0.715	0.916	0.841	0.743	0.952	0.841	0.159	0.031
**2**	0.923	34	0.727	0.618	0.854	0.788	0.671	0.927	0.937	0.210	0.063
**3**	0.870	19	0.567	0.444	0.723	0.652	0.511	0.832	0.827	0.336	0.097
**4**	0.818	11	0.423	0.292	0.613	0.517	0.357	0.748	0.792	0.454	0.123

T: annual interval, survival in the corresponding year. 0: start of study; 1 year: survival up to that year; etc.; ES: expected survival; No at risk: number of individuals at risk at the at the start of the interval; OS: overall survival; 95%CI OS: lower and upper limit of the 95% confidence interval of overall survival; RS: relative survival; 95%CI RS: lower and upper limit of the 95% confidence interval of relative survival; SRI: relative survival in the annual interval; PHF: Probability of dying from hip fracture; POC: Probability of dying from other causes.

Supplementary Data S6. Long-term survival and probability of death due to hip fracture in patients 85 years of age and older.

PRIMARY FEMUR FRACTURE

T	ES	No. at risk	OS	95%CI OS		RS	95%CI RS		SRI	PHF	POC
0	1.000	1433	1	1	1	1	1	1	1	0	0
1	0.879	867	0.684	0.660	0.708	0.778	0.751	0.806	0.778	0.222	0.094
2	0.766	643	0.565	0.539	0.593	0.738	0.704	0.774	0.949	0.257	0.178
3	0.658	438	0.445	0.418	0.474	0.676	0.635	0.720	0.916	0.304	0.251
4	0.558	269	0.316	0.290	0.346	0.566	0.518	0.619	0.837	0.377	0.307

CONTRALATERAL FEMUR FRACTURE

T	ES	No. at risk	OS	95%CI OS		RS	95%CI RS		SRI	PHF	POC
0	1.000	112	1	1	1	1	1	1	1	0	0
1	0.886	73	0.690	0.608	0.781	0.779	0.688	0.883	0.779	0.221	0.089
2	0.767	50	0.527	0.440	0.632	0.687	0.573	0.823	0.881	0.303	0.170
3	0.656	26	0.351	0.267	0.462	0.535	0.406	0.704	0.778	0.420	0.229
4	0.583	20	0.293	0.211	0.405	0.503	0.363	0.696	0.940	0.441	0.266

T: annual interval, survival in the corresponding year. 0: start of study; 1 year: survival up to that year; etc.; ES: expected survival; No at risk: number of individuals at risk at the at the start of the interval; OS: overall survival; 95%CI OS: lower and upper limit of the 95% confidence interval of overall survival; RS: relative survival; 95%CI RS: lower and upper limit of the 95% confidence interval of relative survival; SRI: relative survival in the annual interval; PHF: Probability of dying from hip fracture; POC: Probability of dying from other causes.

Supplementary Data S7. Long-term survival and probability of death due to hip fracture in patients with normal cognitive functioning (0–2 errors in Short Portable Mental Status Questionnaire).

PRIMARY FEMUR FRACTURE

T	ES	No. at risk	OS	95%CI OS		RS	95%CI RS		SRI	PHF	POC
0	1.000	1191	1	1	1	1	1	1	1	0	0
1	0.930	921	0.849	0.829	0.870	0.913	0.892	0.936	0.913	0.087	0.064
2	0.859	757	0.770	0.746	0.795	0.896	0.867	0.925	0.981	0.103	0.127
3	0.791	586	0.687	0.659	0.715	0.869	0.834	0.906	0.970	0.126	0.187
4	0.723	428	0.583	0.552	0.616	0.806	0.764	0.851	0.928	0.176	0.241

CONTRALATERAL FEMUR FRACTURE

T	ES	No. at risk	OS	95%CI OS		RS	95%CI RS		SRI	PHF	POC
0	1.000	60	1	1	1	1	1	1	1	0	0
1	0.923	49	0.865	0.782	0.956	0.937	0.848	1.000	0.937	0.063	0.072
2	0.842	37	0.771	0.669	0.888	0.916	0.795	1.000	0.977	0.083	0.146
3	0.761	23	0.610	0.489	0.762	0.802	0.643	1.000	0.876	0.179	0.212
4	0.700	13	0.509	0.377	0.687	0.727	0.538	0.981	0.906	0.236	0.255

T: annual interval, survival in the corresponding year. 0: start of study; 1 year: survival up to that year; etc.; ES: expected survival; No at risk: number of individuals at risk at the at the start of the interval; OS: overall survival; 95%CI OS: lower and upper limit of the 95% confidence interval of overall survival; RS: relative survival; 95%CI RS: lower and upper limit of the 95% confidence interval of relative survival; SRI: relative survival in the annual interval; PHF: Probability of dying from hip fracture; POC: Probability of dying from other causes.

Supplementary Data S8. Long-term survival and probability of death due to hip fracture in patients with mild cognitive impairment (3–4 errors in Short Portable Mental Status Questionnaire).

PRIMARY FEMUR FRACTURE

T	ES	No. at risk	OS	95%CI OS		RS	95%CI RS		SRI	PHF	POC
**0**	1.000	303	1	1	1	1	1	1	1	0	0
**1**	0.907	208	0.733	0.684	0.785	0.808	0.754	0.865	0.808	0.192	0.075
**2**	0.817	154	0.583	0.529	0.643	0.714	0.647	0.788	0.884	0.277	0.140
**3**	0.734	125	0.516	0.460	0.578	0.703	0.628	0.788	0.985	0.286	0.198
**4**	0.650	87	0.401	0.345	0.466	0.617	0.531	0.716	0.877	0.349	0.250

CONTRALATERAL FEMUR FRACTUR

T	ES	No. at risk	OS	95%CI OS		SR	95%CI RS		SRI	PHF	POC
**0**	1.000	24	1	1	1	1	1	1	1	0	0
**1**	0.915	18	0.789	0.644	0.967	0.862	0.703	1.000	0.862	0.138	0.073
**2**	0.852	15	0.658	0.495	0.875	0.772	0.580	1.000	0.896	0.220	0.122
**3**	0.716	9	0.567	0.400	0.803	0.792	0.559	1.000	1.000	0.220	0.213
**4**	0.640	6	0.378	0.217	0.658	0.591	0.340	1.000	0.746	0.364	0.258

T: annual interval, survival in the corresponding year. 0: start of study; 1 year: survival up to that year; etc.; ES: expected survival; No at risk: number of individuals at risk at the at the start of the interval; OS: overall survival; 95%CI OS: lower and upper limit of the 95% confidence interval of overall survival; RS: relative survival; 95%CI RS: lower and upper limit of the 95% confidence interval of relative survival; SRI: relative survival in the annual interval; PHF: Probability of dying from hip fracture; POC: Probability of dying from other causes.

Supplementary Data S9. Long-term survival and probability of death due to hip fracture in patients with moderate cognitive impairment (5–7 errors in Short Portable Mental Status Questionnaire).

PRIMARY FEMUR FRACTURE

T	ES	No. at risk	OS	95%CI OS		RS	95%CI RS		SRI	PHF	POC
**0**	1.000	377	1	1	1	1	1	1	1	0	0
**1**	0.901	215	0.670	0.624	0.721	0.744	0.692	0.800	0.744	0.256	0.074
**2**	0.809	152	0.545	0.494	0.601	0.674	0.610	0.743	0.905	0.320	0.135
**3**	0.722	98	0.405	0.353	0.465	0.561	0.488	0.644	0.833	0.411	0.184
**4**	0.647	58	0.280	0.230	0.340	0.433	0.357	0.527	0.773	0.503	0.217

CONTRALATERAL FEMUR FRACTURE

T	ES	No. at risk	OS	95%CI OS		SR	95%CI RS		SRI	PHF	POC
**0**	1.000	35	1	1	1	1	1	1	1	0	0
**1**	0.921	25	0.738	0.607	0.898	0.801	0.659	0.975	0.801	0.199	0.063
**2**	0.858	17	0.573	0.427	0.768	0.668	0.498	0.895	0.833	0.322	0.105
**3**	0.754	8	0.387	0.247	0.606	0.513	0.328	0.804	0.768	0.455	0.158
**4**	0.663	5	0.276	0.148	0.515	0.416	0.224	0.775	0.811	0.528	0.196

T: annual interval, survival in the corresponding year. 0: start of study; 1 year: survival up to that year; etc.; ES: expected survival; No at risk: number of individuals at risk at the at the start of the interval; OS: overall survival; 95%CI OS: lower and upper limit of the 95% confidence interval of overall survival; RS: relative survival; 95%CI RS: lower and upper limit of the 95% confidence interval of relative survival; SRI: relative survival in the annual interval; PHF: Probability of dying from hip fracture; POC: Probability of dying from other causes.

Supplementary Data S10. Long-term survival and probability of death due to hip fracture in patients with severe cognitive impairment (8 or more errors in Short Portable Mental Status Questionnaire).

PRIMARY FEMUR FRACTURE

T	ES	No. at risk	OS	95%CI OS		RS	95%CI RS		SRI	PHF	POC
0	1.000	596	1	1	1	1	1	1	1	0	0
1	0.902	309	0.601	0.563	0.642	0.666	0.623	0.711	0.666	0.334	0.065
2	0.818	216	0.457	0.417	0.501	0.559	0.510	0.613	0.839	0.431	0.112
3	0.730	143	0.324	0.286	0.368	0.444	0.391	0.504	0.794	0.525	0.151
4	0.651	95	0.237	0.202	0.279	0.364	0.310	0.429	0.821	0.583	0.180

CONTRALATERAL FEMUR FRACTURE

T	ES	No. at risk	OS	95%CI OS		RS	95%CI RS		SRI	PHF	POC
0	1.000	56	1	1	1	1	1	1	1	0	0
1	0.900	27	0.558	0.442	0.706	0.620	0.491	0.784	0.620	0.380	0.062
2	0.800	18	0.407	0.293	0.566	0.509	0.367	0.708	0.821	0.480	0.113
3	0.707	8	0.222	0.131	0.376	0.314	0.185	0.532	0.617	0.636	0.142
4	0.707	8	0.222	0.131	0.376	0.314	0.185	0.532	1.000	0.636	0.142

T: annual interval, survival in the corresponding year. 0: start of study; 1 year: survival up to that year; etc.; ES: expected survival; No at risk: number of individuals at risk at the at the start of the interval; OS: overall survival; 95%CI OS: lower and upper limit of the 95% confidence interval of overall survival; RS: relative survival; 95%CI RS: lower and upper limit of the 95% confidence interval of relative survival; SRI: relative survival in the annual interval; PHF: Probability of dying from hip fracture; POC: Probability of dying from other causes.

Supplementary Data S11. Long-term survival and probability of death due to hip fracture in patients with independence for basic Activities of Daily Living (score 100 in Barthel Index).

PRIMARY FEMUR FRACTURE

T	ES	No. at risk	OS	95%CI OS		RS	95%CI RS		SRI	PHF	POC
**0**	1.000	496	1	1	1	1	1	1	1	0	0
**1**	0.947	405	0.907	0.881	0.933	0.958	0.931	0.986	0.958	0.042	0.051
**2**	0.891	351	0.837	0.804	0.872	0.939	0.902	0.978	0.981	0.059	0.104
**3**	0.835	299	0.775	0.737	0.815	0.928	0.883	0.976	0.988	0.069	0.156
**4**	0.779	244	0.708	0.666	0.754	0.909	0.855	0.967	0.979	0.086	0.206

CONTRALATERAL FEMUR FRACTURE

T	ES	No. at risk	OS	95%CI OS		SR	95%CI RS		SRI	PHF	POC
**0**	1.000	3	1	1	1	1	1	1	1	0	0
**1**	1.000	2	1	1	1	1	1	1	1	0	0
**2**	0.000	1	0	0	0	0.153	0	0.859	0.153	0.847	0.153

T: annual interval, survival in the corresponding year. 0: start of study; 1 year: survival up to that year; etc.; ES: expected survival; No at risk: number of individuals at risk at the at the start of the interval; OS: overall survival; 95%CI OS: lower and upper limit of the 95% confidence interval of overall survival; RS: relative survival; 95%CI RS: lower and upper limit of the 95% confidence interval of relative survival; SRI: relative survival in the annual interval; PHF: Probability of dying from hip fracture; POC: Probability of dying from other causes.

Supplementary Data S12. Long-term survival and probability of death due to hip fracture in patients with slight dependence for basic Activities of Daily Living (score 91–99 in Barthel Index).

PRIMARY FEMUR FRACTURE

T	ES	No. at risk	OS	95%CI OS		RS	95%CI RS		SRI	PHF	POC
0	1.000	195	1	1	1	1	1	1	1	0	0
1	0.926	152	0.863	0.816	0.913	0.932	0.880	0.986	0.932	0.068	0.069
2	0.852	131	0.821	0.767	0.878	0.964	0.901	1.000	1.000	0.068	0.111
3	0.776	101	0.734	0.670	0.803	0.946	0.863	1.000	0.980	0.084	0.182
4	0.705	71	0.601	0.527	0.685	0.852	0.747	0.971	0.901	0.157	0.242

CONTRALATERAL FEMUR FRACTURE

T	ES	No. at risk	OS	95%CI OS		SR	95%CI RS		SRI	PHF	POC
0	0.992	9	1	1	1	1	1	1	1	0	0
1	0.979	8	0.778	0.490	1.000	0.794	0.514	1.000	0.886	0.206	0.016
2	0.928	7	0.778	0.470	1.000	0.838	0.566	1.000	1.000	0.206	0.016
3	0.895	6	0.778	0.445	1.000	0.869	0.600	1.000	1.000	0.206	0.016
4	0.668	5	0.622	0.197	1.000	0.932	0.711	1.000	1.000	0.206	0.172

T: annual interval, survival in the corresponding year. 0: start of study; 1 year: survival up to that year; etc.; ES: expected survival; No at risk: number of individuals at risk at the at the start of the interval; OS: overall survival; 95%CI OS: lower and upper limit of the 95% confidence interval of overall survival; RS: relative survival; 95%CI RS: lower and upper limit of the 95% confidence interval of relative survival; SRI: relative survival in the annual interval; PHF: Probability of dying from hip fracture; POC: Probability of dying from other causes

Supplementary Data S13. Long-term survival and probability of death due to hip fracture in patients with moderate dependence for basic Activities of Daily Living (score 61–90 in Barthel Index).

PRIMARY FEMUR FRACTURE

T	ES	No. at risk	OS	95%CI OS		RS	95%CI RS		SRI	PHF	POC
0	1.000	1078	1	1	1	1	1	1	1	0	0
1	0.908	727	0.747	0.722	0.774	0.823	0.794	0.852	0.823	0.177	0.076
2	0.824	544	0.623	0.593	0.654	0.756	0.720	0.794	0.919	0.238	0.140
3	0.739	387	0.521	0.490	0.555	0.705	0.662	0.750	0.932	0.280	0.199
4	0.662	252	0.393	0.361	0.428	0.594	0.545	0.647	0.843	0.362	0.245

CONTRALATERAL FEMUR FRACTUR

T	ES	No. at risk	OS	95%CI OS		SR	95%CI RS		SRI	PHF	POC
0	1.000	82	1	1	1	1	1	1	1	0	0
1	0.922	58	0.753	0.665	0.852	0.817	0.722	0.925	0.817	0.183	0.064
2	0.841	43	0.667	0.570	0.780	0.793	0.678	0.928	0.971	0.205	0.128
3	0.757	26	0.567	0.463	0.695	0.749	0.612	0.917	0.944	0.242	0.191
4	0.691	15	0.441	0.328	0.594	0.638	0.474	0.859	0.852	0.326	0.233

T: annual interval, survival in the corresponding year. 0: start of study; 1 year: survival up to that year; etc.; ES: expected survival; No at risk: number of individuals at risk at the at the start of the interval; OS: overall survival; 95%CI OS: lower and upper limit of the 95% confidence interval of overall survival; RS: relative survival; 95%CI RS: lower and upper limit of the 95% confidence interval of relative survival; SRI: relative survival in the annual interval; PHF: Probability of dying from hip fracture; POC: Probability of dying from other causes.

Supplementary Data S14. Long-term survival and probability of death due to hip fracture in patients with severe dependence for basic Activities of Daily Living (score 21–60 in Barthel Index).

PRIMARY FEMUR FRACTURE

T	ES	No. at risk	OS	95%CI OS		RS	95%CI RS		SRI	PHF	POC
**0**	1.000	616	1	1	1	1	1	1	1	0	0
**1**	0.903	325	0.608	0.570	0.649	0.673	0.631	0.718	0.673	0.327	0.065
**2**	0.816	222	0.473	0.433	0.516	0.580	0.531	0.633	0.861	0.412	0.116
**3**	0.728	146	0.343	0.304	0.388	0.471	0.418	0.532	0.813	0.500	0.157
**4**	0.648	88	0.241	0.205	0.284	0.372	0.316	0.438	0.789	0.572	0.187

CONTRALATERAL FEMUR FRACTURE

T	ES	No. at risk	OS	95%CI OS		SR	95%CI RS		SRI	PHF	POC
**0**	1.000	70	1	1	1	1	1	1	1	0	0
**1**	0.903	42	0.662	0.559	0.784	0.733	0.619	0.868	0.733	0.267	0.071
**2**	0.804	31	0.501	0.394	0.637	0.623	0.490	0.792	0.850	0.366	0.133
**3**	0.716	18	0.313	0.217	0.451	0.437	0.303	0.630	0.700	0.517	0.170
**4**	0.637	14	0.272	0.181	0.411	0.427	0.283	0.644	0.979	0.523	0.205

T: annual interval, survival in the corresponding year. 0: start of study; 1 year: survival up to that year; etc.; ES: expected survival; No at risk: number of individuals at risk at the at the start of the interval; OS: overall survival; 95%CI OS: lower and upper limit of the 95% confidence interval of overall survival; RS: relative survival; 95%CI RS: lower and upper limit of the 95% confidence interval of relative survival; SRI: relative survival in the annual interval; PHF: Probability of dying from hip fracture; POC: Probability of dying from other causes.

Supplementary Data S15. Long-term survival and probability of death due to hip fracture in patients with total dependence for basic Activities of Daily Living (score 0–20 in Barthel Index).

PRIMARY FEMUR FRACTURE

T	ES	No. at risk	OS	95%CI OS		RS	95%CI RS		SRI	PHF	POC
**0**	1.000	82	1	1	1	1	1	1	1	0	0
**1**	0.896	43	0.567	0.472	0.682	0.633	0.527	0.760	0.633	0.367	0.066
**2**	0.800	30	0.420	0.326	0.542	0.525	0.407	0.677	0.829	0.464	0.116
**3**	0.676	18	0.252	0.172	0.370	0.373	0.254	0.546	0.710	0.586	0.162
**4**	0.571	14	0.222	0.146	0.338	0.389	0.256	0.592	1.000	0.586	0.192

CONTRALATERAL FEMUR FRACTURE

T	ES	No. at risk	OS	95%CI OS		SR	95%CI RS		SRI	PHF	POC
**0**	1.000	11	1	1	1	1	1	1	1	0	0
**1**	0.983	10	0.909	0.761	1.000	0.925	0.774	1.000	0.925	0.075	0.016
**2**	0.869	6	0.545	0.329	0.905	0.627	0.378	1.000	0.678	0.368	0.087
**3**	0.743	1	0.136	0.037	0.506	0.183	0.049	0.677	0.291	0.754	0.110
**4**	0.743	1	0.136	0.037	0.506	0.183	0.049	0.677	1.000	0.754	0.110

T: annual interval, survival in the corresponding year. 0: start of study; 1 year: survival up to that year; etc.; ES: expected survival; No at risk: number of individuals at risk at the at the start of the interval; OS: overall survival; 95%CI OS: lower and upper limit of the 95% confidence interval of overall survival; RS: relative survival; 95%CI RS: lower and upper limit of the 95% confidence interval of relative survival; SRI: relative survival in the annual interval; PHF: Probability of dying from hip fracture; POC: Probability of dying from other causes
